# Supplementary material for: Knowledge-aware contrastive heterogeneous molecular graph learning
Source: PLoS Comput Biol. 2025 May 12;21(5):e1013008. doi: 10.1371/journal.pcbi.1013008 (PMC12068650; doi:10.1371/journal.pcbi.1013008)
Supplement: S1 Appendix — In the Appendix, we provide a detailed overview of work related to molecular representation learning and include additional experimental details. These include a description of the datasets, the initialization of the two knowledge graphs, feature extraction for nodes and edges in molecular modeling, and parameter selection details. We also present a theoretical proof demonstrating that the contrastive learning loss function effectively optimizes the model [file pcbi.1013008.s001.pdf]

1 Related Work

1.1 Molecular Representation Learning

Molecular representation learning has increasingly been aligned with the Message Passing Neural Network (MPNN) framework as outlined by Gilmer et al. [1]. This approach is complemented by various variants of graph neural networks, such as GCN [2], GIN [3], GAT [4], GGNN [5], GraphSage [6], and HetGNN [7], each of which offers unique perspectives on molecular structure learning. Emphasizing the topology of molecular graphs, MPNN variants such as DMPNN [8], CMPNN [9], and CoMPT [10] exploit node and edge attributes for improved message passing efficiency. The field has also ventured into self-supervised learning, as seen in strategies such as context prediction and attribute masking by PreGNN [11], and GROVER's [12] molecule-specific methods, which include contextual property and graph-level motif prediction. These methods excel at detailing the minute, microscopic aspects of molecules, but often overlook the broader pharmacological context in which these molecules operate. KG-based methods, particularly KGNN [13] and MDNN [14], integrate external knowledge and view molecules as interconnected nodes. They consider factors such as drug-target interactions and therapeutic effects, providing a holistic view of the drug's role in a larger biological context. However, a notable limitation of these methods is their inability to effectively integrate the microscopic attributes of drug molecules with their macroscopic properties.

1.2 Contrastive Learning on Molecular Graphs

Contrastive learning has revolutionized the generalizability and robustness of graph encoders in molecular contexts. Graph augmentation methods such as node dropping, edge perturbation, attribute masking, and subgraph creation, as proposed by You et al. [15], have proven effective in molecular contexts. InfoGraph [16] advances this by maximizing the mutual information between the global representations of the graph and its various substructures. In a departure from traditional methods, models such as CKGNN [17] use chemical domain knowledge to select positive pairs based on fingerprint similarity. Wang et al. [18] draw inspiration from equivalence relations in chemical reactions. A novel approach, KCL [19], uses contrastive learning with an external KG structured as triples (chemical element, relation, attribute), which enhances molecular learning with rich external knowledge. While the above contrastive learning methods generate new views of molecules, they often overlook the fact that small structural perturbations can lead to significant changes in the pharmacological properties of a molecule. Moreover, despite these advances, most models, including those using external KGs, struggle to account for the nuanced pharmacological properties of molecules, especially when generating negative samples, a critical aspect for accurate molecular differentiation.

2 Experimental details

2.1 Dataset Description

Table 1 provides a comprehensive overview of the statistics related to both the elemental knowledge graph (KG) and the drug knowledge graph (KG), detailing the number of entities, relations, and edges present in each.

Additionally, Table 2 presents the statistical breakdown of the datasets used for the molecule property prediction tasks, including the number of molecules, and the description of tasks. These statistics are crucial for understanding the scope and relevance of the datasets employed in validating the performance of our model across different predictive tasks.

KG	Node	Node type	Edge	Edge type	Node Used
Element	413	3	3,014	24	30(e)+83(f)
Drug	97,238	13	5,874,261	107	8,358(d)

Table 1. Summary of KGs for pre-training in this work. In the column 'Node Used', the letter within parentheses signifies the category of the node: 'e' for an element, 'f' for a functional group, and 'd' for a drug molecule.

Dataset	# Molecules	Description	Task Type	Metric
BBBP	2,039	Binary labels of blood-brain barrier penetration(permeability).	Classification	ROC-AUC
Tox21	7,831	Qualitative toxicity measurements on 12 biological targets	Classification	ROC-AUC
ToxCast	8,575	Toxicology data for a large library of compounds based on in vitro high-throughput screening	Classification	ROC-AUC
ClinTox	1,478	Drugs approved by the FDA and those that have failed clinical trials for toxicity reasons.	Classification	ROC-AUC
HIV	41,127	Experimentally measured abilities to inhibit HIV replication.	Classification	ROC-AUC
BACE	1,513	Quantitative (IC50) and qualitative binding results for a set of inhibitors of BACE-1.	Classification	ROC-AUC
SIDER	1,427	Database of marketed drugs and adverse drug reactions (ADR)	Classification	ROC-AUC
MUV	93,087	Subset of PubChem BioAssay by applying a refined nearest neighbor analysis	Classification	ROC-AUC
FreeSolv	642	Experimental and calculated hydration free energy of small molecules in water.	Regression	RMSE
ESOL	1,128	Water solubility data(log solubility in mols per litre) for common organic small molecules.	Regression	RMSE
Lipo	4,200	Experimental results of octanol/water distribution coefficient	Regression	RMSE
QM7	6,830	Electronic properties determined using ab-initio density functional theory(DFT).	Regression	MAE
QM8	21,786	Electronic spectra and excited state energy calculated by multiple quantum mechanic methods.	Regression	MAE

Table 2. Summary of all the benchmarks for molecular property predictions.

Table 3 presents the statistical breakdown of the TwoSide datasets used for the DDI prediction tasks, including the number of molecules, and the description of tasks.

Name	Drug	Interactions	Positive DDI Tuples	Negative Ratio
TwoSide	645	963	4576,287	1.0

Table 3. Summary of TwoSide Dataset.

2.2 Knowledge Initialization

Hierarchical Elemental KG Initialization: We initialize the elemental KG using HAKE [20], where entities are mapped into polar coordinate systems. This provides a strong geometric basis for representing the relationships between elements and functional groups. The equation is given by:

$$f_r(\mathbf{h},\mathbf{t}) = -\|\mathbf{h}_m \circ \mathbf{r}_m - \mathbf{t}_m\|_2 - \lambda \| \sin((\mathbf{h}_p + \mathbf{r}_p - \mathbf{t}_p)/2)\|_1 \quad (1)$$

Here, $\mathbf{h}_m$ and $\mathbf{t}_m$ represent the moduli of the head and tail entities, while $\mathbf{h}_p$ and $\mathbf{t}_p$ denote their phases. The transformations $\mathbf{r}_m$ and $\mathbf{r}_p$ capture the relationships between these entities. This embedding ensures that molecules with similar structural components are closely related in the embedding space.

Drug KG Initialization: For drug-related molecules, we employ a TransE-based pre-training scheme that captures relationships between drug entities. This is particularly effective in initializing the embeddings for the D-nodes. The scoring function is given as:

$$f_r(\mathbf{h},\mathbf{t}) = -\|\mathbf{h} + \mathbf{r} - \mathbf{t}\|_{1/2} \quad (2)$$

2.3 Feature Extraction for Atoms and Fragments

We use the chemical characteristics of atoms and fragments in molecules to initialize the **node representation** to obtain the initial representation of some nodes and edges in HMG. The feature extraction by RDKIT contains four parts: 1) Atom feature extraction, 2) Atom Bond feature extraction, 3) Fragment feature extraction, and 4) Fragment Bond feature extraction. We use RDKit to extract all features as the input of KCHML. Table 4, 5, 6 and 7 show the features we used in KCHML.

Table 4. Atom Features

Features	Size	Description
Atomic Number	16	element number in the periodic table (e.g 5 for Carbon)
Total Degree	8	degree of the atom in the molecule including Hs
Degree	8	degree of the atom in the molecule excluding Hs.
Formal Charge	8	hypothetical charge assigned to an atom
Chirality	4	chirality of the atom
Number of H	6	number of bond hydrogen atoms
Hybridization	6	sp, sp2, sp3, sp3d, sp3d2 or unknown
Total Valence	8	number of valences of the atom
Idx	1	index of the atom in the molecule
Mass	1	mass of the atom
Aromatic	1	1 if this atom is part of an aromatic system else 0
In-ring	1	1 if the atom is part of a ring else 0
Total	68	

Table 5. Atom Bond Features

Features	Size	Description
Existence	1	0 if the bond exists else 1
Bond Type	5	single, double, triple, aromatic or unknown
Stereo	7	none, any, E/Z or cis/trans or unknown
Conjugated	1	1 if the bond is considered to be conjugated else 0
Aromatic	1	1 if the bond is part of an aromatic system else 0
In-ring	1	1 if the bond is part of a ring else 0
Total	16	

Table 6. Fragment Features

Features	Size	Description
MACCS Key	167	chemical fingerprinting of the fragment.
Factory Type	27	features of the fragment in SMARTS dictionary
Total	194	

Table 7. Fragment Bond Features

Features	Size	Description
Breakpoint 1	17	The type of one breakpoint after the BRICS algorithm.
Breakpoint 2	17	The type of another breakpoint after the BRICS algorithm.
Total	34	

2.3.1 Implementation details

Our model is implemented using PyTorch [21] and the Deep Graph Library [17], with RDKit employed for the extraction of fragments and functional groups. The Adam optimizer [22] is utilized, initialized with a learning rate lr of 0.0001. The temperature parameter τ is set to 0.1, as recommended in [23].

During the pre-training phase, a batch size that is too small could lead to inadequate utilization of molecules with drug IDs, as only those drug molecules most similar to the cluster center would be included in the batch. To address this, considering the ratio of molecules with drug IDs to those without and the total number of drug molecules to be included, we opted for a batch size of 256 and a fixed number of 50 epochs.

We evaluated embedding sizes of 64, 128, and 256. The performance improvement with larger embedding sizes was marginal, leading us to select an embedding size of 128 for both input and output embeddings, as it offered the most balanced trade-off between model complexity and task performance.

The model configuration includes 8 attention heads. For downstream tasks, early stopping is employed based on validation set performance, and hyperparameters are optimized using random search based on validation set outcomes. All code development and experiments were conducted on an Ubuntu server equipped with two NVIDIA GeForce 4090 GPUs.

3 Proofs

We believe that calculating the loss between any two views in this way can effectively optimize the model, as stated below in Theorem 1:

Theorem 1. *Minimizing the loss (10) maximizes a lower bound on mutual information between positive pairs.*

To obtain the above conclusion, we remodeled the loss calculation process. Given a molecular graph a, we constructed a sample set $X = \{x_1, \ldots x_N\}$ of N random samples, where there is 1 positive sample x_t and $N-1$ negative samples. Then, a discriminant function $\delta_\theta(\cdot)$ was trained to achieve a high value for positive pairs (x_t, a) and low for negative pairs. In this study, $\delta_\theta(\cdot)$ in equation (9) is implemented in the following form:

$$\delta_\theta(\mathcal{G}_1, \mathcal{G}_2) = exp(\frac{\mathbf{z}_{\mathcal{G}_1} \cdot \mathbf{z}_{\mathcal{G}_2}}{||\mathbf{z}_{\mathcal{G}_1}|| \cdot ||\mathbf{z}_{\mathcal{G}_2}||} \cdot \frac{1}{\tau}). \tag{3}$$

Contrastive learning aims to create an embedding space where samples from distinct distributions are distinguishable. Typically, the probability of a positive sample x_t is sampled from the conditional probability $p(x_t|a)$, while the negative sample is from the suggested distribution $p(x_i)$, since it is independent of a. Therefore, optimizing loss function can be regarded as enhancing optimizing conditional probability $p(x_t|X, a)$.

Lemma 1. *The optimal discriminant function $\delta^*_\theta(x_t, a)$ is proportional to the density ratio:*

$$\delta^*_\theta(x_t, a) \propto \frac{p(x_t|a)}{p(x_t)} \tag{4}$$

Proof of Lemma 1. By merging the two views' samples, each term in the loss function (10) can be written as:

$$\mathcal{L} = -\mathop{\mathbb{E}}_X \left[\log \frac{\delta_\theta(x_t, a)}{\sum_{x_j \in X} \delta_\theta(x_j, a)} \right]. \tag{5}$$

The probability that x_t is a positive sample $p(x_t|X,a)$ can be calculated using the Bayesian probability formula:

$$
\begin{aligned}
p(x_t|X,a) &= \frac{p(x_t|a)\prod_{i\neq t} p(x_i)}{\sum_{j=1}^{N} p(x_j|a)\prod_{i\neq j} p(x_i)} \\
&= \frac{\frac{p(x_t|a)}{p(x_t)}}{\sum_{j=1}^{N}\frac{p(x_j|a)}{p(x_j)}}
\end{aligned} \tag{6}
$$

where we divide $\prod_{i=1}^{N} p(x_i)$ for the numerator and denominator. By comparing the above with equation (5), we observe that the discriminant function $\delta_\theta^*(x_t,a)$ is proportional to the density ratio $\frac{p(x_t|a)}{p(x_t)}$. □

Lemma 2. *Mutual information $I(x_t,a) \geq \log(N) - \mathcal{L}^{opt}$.*

Proof of Lemma 2. By inserting formula (4) back in to equation (5), we derive:

$$
\begin{aligned}
\mathcal{L}^{opt} &= -\mathop{\mathbb{E}}_{X}\log\left[\frac{\frac{p(x_t|a)}{p(x_t)}}{\sum_{j=1}^{N}\frac{p(x_j|a)}{p(x_j)}}\right] \\
&= \mathop{\mathbb{E}}_{X}\log\left[1+\frac{p(x_t)}{p(x_t|a)}\sum_{j\neq t}\frac{p(x_j|a)}{p(x_j)}\right] \\
&\approx \mathop{\mathbb{E}}_{X}\log\left[1+\frac{p(x_t)}{p(x_t|a)}(N-1)\mathop{\mathbb{E}}_{x_j}\frac{p(x_j|a)}{p(x_j)}\right] \\
&= \mathop{\mathbb{E}}_{X}\log\left[1+\frac{p(x_t)}{p(x_t|a)}(N-1)\right] \\
&\geq \mathop{\mathbb{E}}_{X}\log\left[\frac{p(x_t)}{p(x_t|a)}N\right] \\
&= -I(x_t,a)+\log(N).
\end{aligned} \tag{7}
$$

□

Therefore, $I(x_t,a) \geq \log(N) - \mathcal{L}^{opt}$. As the N increases, the approximation step becomes more accurate. Furthermore, given a fixed N, minimizing $\mathcal{L}^{opt}$ maximizes the lower bound on the mutual information $I(x_t,a)$. We have theoretically demonstrated that constructing sample pairs across multiple views can effectively enhance the optimization of the model.

References

1. Gilmer J, Schoenholz SS, Riley PF, Vinyals O, Dahl GE. Neural message passing for quantum chemistry. In: International conference on machine learning. PMLR; 2017. p. 1263–1272.

2. Kipf TN, Welling M. Semi-supervised classification with graph convolutional networks. arXiv preprint arXiv:160902907. 2016;.

3. Xu K, Hu W, Leskovec J, Jegelka S. How powerful are graph neural networks? arXiv preprint arXiv:181000826. 2018;.

4. Velickovic P, Cucurull G, Casanova A, Romero A, Lio P, Bengio Y, et al. Graph attention networks. stat. 2017;1050(20):10–48550.

5. Li Y, Zemel R, Brockschmidt M, Tarlow D. Gated Graph Sequence Neural Networks. In: Proceedings of ICLR'16; 2016.

6. Hamilton W, Ying Z, Leskovec J. Inductive representation learning on large graphs. Advances in neural information processing systems. 2017;30.

7. Zhang C, Song D, Huang C, Swami A, Chawla NV. Heterogeneous graph neural network. In: Proceedings of the 25th ACM SIGKDD international conference on knowledge discovery & data mining; 2019. p. 793–803.

8. Yang K, Swanson K, Jin W, Coley C, Eiden P, Gao H, et al. Analyzing Learned Molecular Representations for Property Prediction. Journal of Chemical Information and Modeling. 2019;59(8):3370.

9. Song Y, Zheng S, Niu Z, Fu ZH, Lu Y, Yang Y. Communicative representation learning on attributed molecular graphs. In: IJCAI International Joint Conference on Artificial Intelligence. vol. 2021. International Joint Conferences on Artificial Intelligence; 2020. p. 2831–2838.

10. Chen J, Zheng S, Song Y, Rao J, Yang Y. Learning Attributed Graph Representation with Communicative Message Passing Transformer. In: Zhou ZH, editor. Proceedings of the Thirtieth International Joint Conference on Artificial Intelligence, IJCAI-21. International Joint Conferences on Artificial Intelligence Organization; 2021. p. 2242–2248. Available from: `https://doi.org/10.24963/ijcai.2021/309`.

11. Hu W, Liu B, Gomes J, Zitnik M, Liang P, Pande V, et al. Strategies For Pre-training Graph Neural Networks. In: International Conference on Learning Representations (ICLR); 2020.

12. Rong Y, Bian Y, Xu T, Xie W, Wei Y, Huang W, et al. Self-supervised graph transformer on large-scale molecular data. Advances in Neural Information Processing Systems. 2020;33:12559–12571.

13. Lin X, Quan Z, Wang ZJ, Ma T, Zeng X. KGNN: Knowledge Graph Neural Network for Drug-Drug Interaction Prediction. In: IJCAI. vol. 380; 2020. p. 2739–2745.

14. Lyu T, Gao J, Tian L, Li Z, Zhang P, Zhang J. MDNN: A Multimodal Deep Neural Network for Predicting Drug-Drug Interaction Events. In: IJCAI; 2021. p. 3536–3542.

15. You Y, Chen T, Sui Y, Chen T, Wang Z, Shen Y. Graph contrastive learning with augmentations. Advances in neural information processing systems. 2020;33:5812–5823.

16. Sun FY, Hoffman J, Verma V, Tang J. InfoGraph: Unsupervised and Semi-supervised Graph-Level Representation Learning via Mutual Information Maximization. In: International Conference on Learning Representations; 2019.

17. Fang Y, Yang H, Zhuang X, Shao X, Fan X, Chen H. Knowledge-aware contrastive molecular graph learning. arXiv preprint arXiv:210313047. 2021;.

18. Wang H, Li W, Jin X, Cho K, Ji H, Han J, et al. CHEMICAL-REACTION-AWARE MOLECULE REPRESENTATION LEARNING. In: 10th International Conference on Learning Representations, ICLR 2022; 2022.

19. Fang Y, Zhang Q, Yang H, Zhuang X, Deng S, Zhang W, et al. Molecular contrastive learning with chemical element knowledge graph. In: Proceedings of the AAAI Conference on Artificial Intelligence. vol. 36; 2022. p. 3968–3976.

20. Zhang Z, Cai J, Zhang Y, Wang J. Learning Hierarchy-Aware Knowledge Graph Embeddings for Link Prediction; 2022.

21. Paszke A, Gross S, Massa F, Lerer A, Bradbury J, Chanan G, et al.. PyTorch: An Imperative Style, High-Performance Deep Learning Library; 2019.

22. Kingma DP, Ba J. Adam: A method for stochastic optimization. arXiv preprint arXiv:14126980. 2014;.

23. Hu W, Liu B, Gomes J, Zitnik M, Liang P, Pande V, et al. Strategies For Pre-training Graph Neural Networks. In: International Conference on Learning Representations (ICLR); 2020.
